# Chloroquine: Rapidly withdrawing from first-line treatment of COVID-19

**DOI:** 10.1016/j.heliyon.2024.e37098

**Published:** 2024-08-28

**Authors:** Yunlong Jia, Wenjie Tian, Yuyao Li, Yuyan Teng, Xiaolin Liu, Zhengyu Li, Mingsheng Zhao

**Affiliations:** Institute of Immunology and Molecular Medicine, Jining Medical University, Jining, China

**Keywords:** CQ, COVID-19, ACE2, Complications, Disorders, Toxity

## Abstract

The COVID-19 outbreak has garnered significant global attention due to its impact on human health. Despite its relatively low fatality rate, the virus affects multiple organ systems, resulting in various symptoms such as palpitations, headaches, muscle pain, and hearing loss among COVID-19 patients and those recovering from the disease. These symptoms impose a substantial physical, psychological, and social burden on affected individuals. On February 15, 2020, the Chinese government advised incorporating antimalarial drugs into the guidelines issued by the National Health Commission of China for preventing, diagnosing, and treating COVID-19 pneumonia. We examine the adverse effects of Chloroquine (CQ) in treating COVID-19 complications to understand why it is no longer the primary treatment for the disease.

## 1 Introduction

COVID-19, a highly contagious respiratory illness, stems from Severe Acute Respiratory Syndrome Coronavirus-2 (SARS-CoV-2), a new virus identified in December 2019 [1]. By early 2020, the World Health Organization recognized the COVID-19 outbreak as a global health emergency, officially declaring it a pandemic on March 11, 2020 [2]. While most individuals infected with SARS-CoV-2 experience mild or no symptoms, some suffer severe illness and may succumb to COVID-19-related complications [[3], [4], [5]].

COVID-19 presents with systemic inflammation, cytokine storm [6], multi-organ dysfunctions, and immune response dysregulation. On one hand, SARS-CoV-2 infection can trigger uncontrolled inflammatory responses by activating both innate and adaptive immune responses [7]. This activation prompts activated macrophages to produce various pro-inflammatory cytokines like Tumor Necrosis Factor (TNF)-α, Interleukin (IL)-1, IL-6, and IL-18, potentially setting off an inflammatory cascade that leads to cytokine storms [8]. On the other hand, SARS-CoV-2 utilizes Angiotensin Converting Enzyme 2 (ACE2) receptors on host cell surfaces for entry [9,10]. The strong expression of ACE2 receptors and increased release of proprotein convertase contribute to COVID-19-related complications by facilitating viral entry into host cells [10,11]. These characteristics can result in patients developing chronic issues and multi-system complications such as cardiovascular injuries, neuropsychiatric manifestations, myositis, retinopathy, and hearing loss [[12], [13], [14], [15], [16]]. Although initial small-scale studies during the early stages of the pandemic showed promise for Chloroquine (CQ) [[17], [18], [19]], the Food and Drug Administration (FDA) revoked Emergency Use Authorization (EUA) on June 15, 2020, and issued treatment guidelines advising against the use of CQ with or without azithromycin for COVID-19 [[20], [21], [22]].

CQ, a derivative of 4-aminoquinoline, has been extensively utilized for nearly eight decades to prevent and treat malaria. Moreover, CQ is commonly employed to alleviate acute and chronic inflammatory conditions such as rheumatoid arthritis, systemic lupus erythematosus, sarcoidosis, and cancer [[23], [24], [25], [26]].

Our research has revealed that CQ, an antimalarial medication, can effectively combat SARS-CoV-2 infection through various mechanisms. In vitro experiments have demonstrated that low concentrations of CQ can impede COVID-19 infection [27]. Additionally, CQ has shown the capability to obstruct viral entry into cells by increasing the endosomal Potential of Hydrogen (PH) to hinder the endocytic pathway [28], and by interfering with the glycosylation of ACE2 receptors [29], thereby reducing the efficiency of the ACE2-SARS-CoV-2 interaction. Furthermore, CQ possesses immunomodulatory properties that mitigate the inflammatory complications associated with viral diseases by inhibiting the production/release of IL-6 [30], which may synergistically enhance its antiviral effects in vivo.

**Fig. 1:**
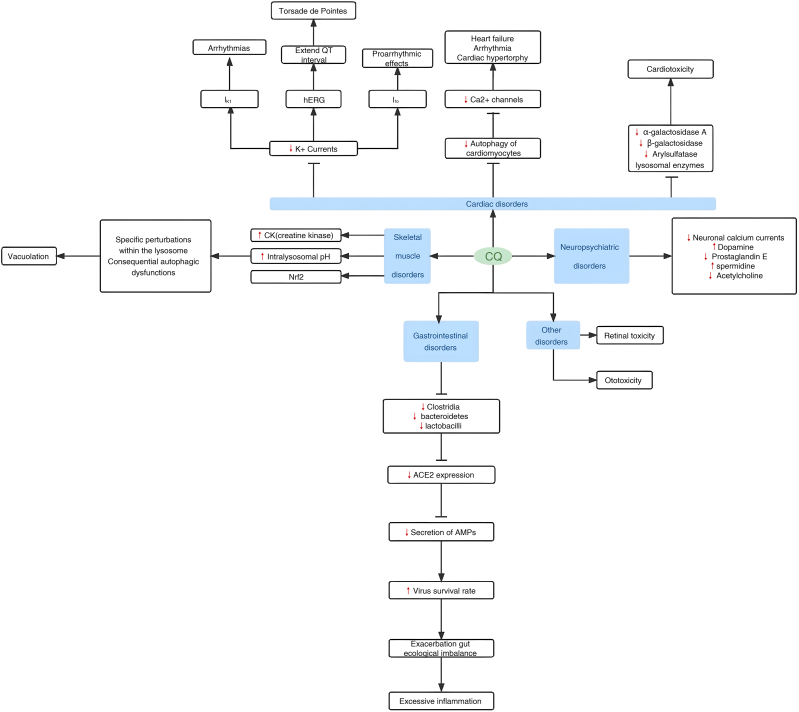
The main mechanism of CQ on the complications of the COVID-19. The adverse effects of CQ encompass the following five categories: CQ inhibits various K+ currents and autophagy processes, which may ultimately result in cardiac disorders; Skeletal muscle symptoms are correlated with elevated levels of Creatine Kinase (CK), lysosomal pH, and the chronic activation of Nrf2; CQ induces neuropsychiatric side effects by impeding neuronal calcium currents, promoting dopamine excess, antagonizing prostaglandin E, causing spermidine excess, and disrupting acetylcholine function; The ecological disturbances provoked by CQ can lead to excessive inflammation, potentially leading to gastrointestinal disorders; Additionally, CQ exhibits ototoxic and retinal toxic effects.

Despite the established effectiveness of CQ in COVID-19 treatment, patients undergoing this therapy may face increased susceptibility to adverse effects (Fig. 1). Notably, the most severe complications include QTc prolongation and cardiac arrhythmia [31]. Furthermore, common drug-related adverse effects encompass nausea, headache, myopathy, retinopathy, and hearing loss. Therefore, this review offers a comprehensive overview of the latest available data on COVID-19-specific complications encountered by patients treated with CQ, with particular emphasis on the mechanisms underlying CQ-induced complications, and to deliberate on the reasons behind the withdrawal of CQ from first-line treatment options for COVID-19.

## 2 Cardiac disorders

COVID-19 can exert a profound influence on the cardiovascular system. Research suggests that certain COVID-19 patients may encounter cardiovascular harm, including endothelial cell damage, cardiac arrhythmias, myocarditis, myocardial interstitial fibrosis, and thrombotic incidents [[32], [33], [34], [35], [36]]. Although not all individuals will manifest these symptoms, healthcare professionals should exercise utmost vigilance regarding COVID-19's possible impact on cardiac wellbeing. Evidence indicates that cardiomyocytes, endothelial cells, and pericytes within the heart possess ACE2 expression, rendering them potential direct targets of SARS-CoV-2 [37].

Initially authorized by the FDA for emergency use in the prophylaxis and/or treatment of COVID-19, CQ medications have been associated with an elevated risk of TdP and QTc prolongation, as well as ventricular arrhythmias in COVID-19 patients. Research indicates that approximately 10 % of COVID-19 patients treated with CQ experience QT prolongation [38]. In comparison to a placebo, one study demonstrated that a dosage of 1000 mg/day of CQ phosphate for three days led to an increase in QTc of 18.4–35 ms by day three. Furthermore, a retrospective analysis conducted in Brazil found that COVID-19 patients treated with CQ exhibited prolonged hospital stays and a higher incidence of QT interval prolongation [39]. Additionally, our investigation revealed clear evidence of ventricular arrhythmias in two out of 28 COVID-19 patients who received high-dose CQ treatment [40].

The cardiovascular adverse effects induced by CQ primarily involve disruptions in K^+^ currents and the autophagy process of cardiomyocytes. The functionality of K^+^ channels plays a crucial role in the repolarization of cardiac myocytes. CQ has been shown to hinder various K^+^ currents, including the inward rectifier K^+^ (Kir) current (IK1) [41], the rapidly activating delayed rectifier K^+^ current (IKr) associated with the human Ether-a-go-go-related Gene (hERG) [42,43], the fast transient outward K^+^ current (Ito) [44], and the ATP-sensitive inward rectifier K^+^ current (KATP) [45,46]. Intriguingly, inhibiting the cardiac IK1 current has been demonstrated to provoke fatal ventricular arrhythmias [41]. Additionally, the inhibition of hERG current has been linked to QT interval prolongation, potentially precipitating Torsades de Pointes under certain circumstances [47]. These findings suggest that CQ acts as an open channel blocker of the Ito current, potentially inducing proarrhythmic effects in clinical scenarios.

While there is substantial evidence suggesting that autophagy activation may offer benefits in cases of myocardial infarction/reperfusion and ischemic heart disease by safeguarding cardiomyocytes against necroptosis and apoptosis, as well as thwarting myocardial fibrosis and hypertrophy, treatment with CQ could counteract these protective effects by inhibiting autophagic flux [[48], [49], [50], [51], [52], [53], [54], [55], [56]]. CQ has been demonstrated to suppress autophagy, potentially resulting in the inhibition of Ca^2+^ channels and, consequently, precipitating cardiac disorders such as arrhythmia, cardiac hypertrophy, and heart failure [57]. Moreover, studies have suggested that CQ may induce cardiotoxicity by impeding lysosomal enzymes, including α-galactosidase A, β-galactosidase, and arylsulfatase in cardiomyocytes [58]. Intriguingly, CQ can also influence mitochondrial respiratory chain complexes to elevate levels of ROS and oxidative stress, ultimately instigating heart disease by initiating mitochondrial apoptosis signaling in heart cardiomyocytes [59]. The FDA subsequently revoked the EUA, possibly due to the excessive cardiovascular side effects associated with CQ. Hence, the use of CQ should be cautiously weighed against its anticipated therapeutic benefits and administered under close monitoring.

## 3 Skeletal muscle disorders

Skeletal muscle symptoms and elevated levels of Creatine Kinase (CK) are frequently observed in individuals affected by COVID-19. Interestingly, studies have established a significant correlation between COVID-19-related skeletal muscle symptoms and signs, and CK levels [60]. CK is released from muscles when there is a disruption of muscle membrane integrity or direct damage to muscular tissue. This often occurs concurrently with an exaggerated inflammatory response, akin to the cytokine release syndrome associated with COVID-19 [60,61].

Recent investigations conducted in China revealed that 44–70 % of hospitalized COVID-19 patients experienced symptoms of myalgia or fatigue, while up to 33 % exhibited elevated CK levels [62,63]. Hence, it is plausible that coronavirus infections can precipitate viral myositis. The occurrence of skeletal muscle injury and myopathy in COVID-19 patients may be linked to increased neutrophil count, decreased lymphocyte count, elevated C-reactive protein levels, and elevated D-dimer levels [60,61]. Rhabdomyolysis is also frequently documented as both an initial presentation and subsequent complication of COVID-19, often associated with elevated inflammatory markers [[64], [65], [66], [67]].

Moreover, the virus may directly infiltrate muscle tissue by binding to the ACE2 receptor expressed on myocytes, leading to the development of myositis [60,68]. Consequently, it has been theorized that COVID-19-induced myopathy could arise as a consequence of the inflammatory cascade [69].

During the initial year of the COVID-19 pandemic, CQ and its derivative Hydroxychloroquine (HCQ) were utilized, but their use has been associated with potential symptoms of myopathy [70]. Administration of CQ may lead to the development of myopathies in individuals, while also affecting CK levels.

A comprehensive narrative review of patients treated with CQ revealed that elevated CK levels were present in 60.7 % of cases, and electromyography data exhibited a myopathic pattern in 54 % of individuals examined [71]. Moreover, muscle biopsies from patients receiving CQ displayed vacuolation in 53.7 % of cases, with curvilinear bodies being the main focus of ultrastructural detection (86.8 %) [71]. The presence of rimmed vacuolar changes in muscle biopsies of patients with antimalarial-induced myopathy is considered the most representative feature observed, as similar pathological features have been confirmed in other neuromuscular disorders such as sporadic and familial inclusion body myositis, oculopharyngeal muscular dystrophy, myofibrillary myopathy, and related myopathies [[72], [73], [74], [75]].

On one hand, CQ is regarded as a weak base due to the presence of a basic side chain, allowing these drugs to accumulate in intracellular compartments, primarily lysosomal organelles. The amphiphilic nature of these compounds contributes to an increase in lysosomal pH, ultimately leading to specific disturbances within the lysosome and subsequent autophagic dysfunctions that result in vacuolar changes in muscle tissue [76]. Additionally, these compounds can specifically inhibit the lysosomal proteinase, cathepsin B, which plays a crucial role in intracellular proteolysis [76]. Importantly, the effects of CQ administration extend beyond peripheral organelles within the cell and appear to involve other signaling pathways as well. One regulatory mechanism involves the transcription factor Nrf2, a critical leucine zipper protein that regulates the expression of antioxidant proteins to mitigate oxidative damage induced by injury and inflammation [77].

In disorders characterized by vacuolar changes affecting muscle function, such as autophagic vacuolar myopathy induced by CQ, chronic activation of Nrf2 can have adverse effects on organ systems. Prolonged activation of Nrf2 in skeletal muscle tissue can lead to alterations in cellular redox balance, thereby contributing to the progression of muscular pathologies [[78], [79], [80]]. In one study, CQ administration was implicated in exacerbating cases of myasthenia gravis [81]. However, despite such findings, there was no significant evidence linking chronic fatigue syndrome with the use of CQ [82]. In conclusion, it is crucial not to overlook the monitoring of skeletal muscles in COVID-19 patients receiving CQ treatment.

## 4 Neuropsychiatric disorders

COVID-19 is linked to an increased risk of developing psychiatric disorders and the potential exacerbation of pre-existing conditions, as indicated by numerous reports. The spectrum of neuropsychiatric manifestations encompasses symptoms such as hyposmia/anosmia, headache, and neuromuscular dysfunction, extending to seizures, stroke, altered mental status, encephalopathy, and psychiatric disorders, both in the acute and chronic phases. These manifestations underscore their potential to signal deteriorating clinical outcomes and a poor prognosis [[83], [84], [85], [86]].

The utilization of CQ, an antimalarial medication, emerged as an emergency measure for treating COVID-19 in late March and early April 2020, but it is associated with neuropsychiatric side effects such as seizures, coma, and psychosis, particularly in patients with no prior history of psychiatric disorders [87]. A patient who underwent treatment with HCQ for COVID-19 infection was reported to have developed depression accompanied by melancholic features, severe suicidal ideation, and attempted suicide [88]. Several mechanisms have been proposed to explain CQ-induced neuropsychiatric side effects, including the inhibition of neuronal calcium currents [89], dopamine excess [90], prostaglandin E antagonism [91], spermidine excess [92], and acetylcholine dysfunction [93], ultimately resulting in neuropsychiatric symptoms, including suicidal ideation.

Moreover, there is substantial evidence indicating that Central Nervous System (CNS) levels of CQ are significantly higher (ten to thirty times) than serum levels [94], potentially due to interference with P-glycoprotein function in the blood-brain barrier [95]. Additionally, existing research suggests that CQ and HCQ may mildly inhibit the metabolic activity of CYP2D6, which is involved in metabolizing psychiatric medications, while medications affecting CYP2D6 or CYP3A4 could contribute to alterations in the levels of CQ and HCQ [96,97].

Although neuropsychiatric symptoms directly linked to CQ usage are relatively rare, research on this matter remains inconclusive. Therefore, awareness of the potential types of neuropsychiatric complications that may arise in patients receiving CQ treatment, especially those in intensive care units with COVID-19 where prompt clinical decision-making is crucial, could have significant beneficial implications.

## 5 Gastrointestinal disorders

CQ is believed to be associated with gastrointestinal disorders caused by COVID-19, primarily due to its impact on the gut microbiota. The relative abundance of Bacteroidetes and Firmicutes, as well as the ratio of Bifidobacteria and Lactobacilli, are crucial factors in maintaining gut balance and overall health [98]. Studies have shown that patients receiving HCQ experience a significant decrease in the number of Bacteroidetes, Firmicutes, and Lactobacillus [99]. The expression of ACE2 in the intestinal tract is regulated by intestinal bacteria. Inhibition of intestinal bacteria can lead to downregulation of ACE2 expression, resulting in reduced secretion of AMPs, increased viral survival, and exacerbation of intestinal ecological imbalance [100]. Notably, viral infections can increase the permeability of the gastrointestinal wall to foreign pathogens, allowing invasion of intestinal cells and leading to malabsorption and diarrhea [101]. Moreover, a significant portion (70–80 %) of all immune cells in the human body are located in the gut-associated lymphoid tissue. The gut microbiome plays a crucial role in regulating immune responses and influencing the severity of COVID-19 infection [102]. Some suggest that the interaction and balance between the gut microbiota and the immune system are critical for gut homeostasis. However, dysbiosis caused by CQ may lead to excessive inflammation, resulting in pathological changes in the disease [98]. Therefore, it is essential to pay attention to the regulation of gastrointestinal microbiota in COVID-19 patients after CQ treatment.

## 6 Other disorders

### 6.1 Toxity

There is evidence suggesting that COVID-19 infection can trigger a state of diabetogenesis. Similar to other acute infections, severe cases of COVID-19 induce a non-specific activation of the immune system, leading to an excessive release of counter-regulatory hormones and pro-inflammatory cytokines such as IL-6 and TNF-α. These molecules are known to induce insulin resistance and hyperglycemia [103]. As more individuals recognize the potential benefits of the low-cost antiviral and anti-inflammatory capabilities associated with CQ and its derivative HCQ, these drugs have garnered attention in the fight against COVID-19 [104]. Furthermore, HCQ has been found to possess oral hypoglycemic properties that can lower blood sugar levels. Sharing an identical mechanism of action with CQ, HCQ differs in structure only by the presence of an additional hydroxy moiety in one terminal [105]. Thus, CQ holds promise as a potential treatment option for diabetic patients with COVID-19 infection. However, current evidence offers limited support for the effectiveness and safety of CQ and HCQ in treating COVID-19 patients with diabetes. Notably, given their anti-COVID-19 mechanisms of action, the administration of CQ phosphate in patients with severe type 2 diabetes carries the risk of causing harm [106]. Retinopathy incidence represents a severe complication and ranks among the major dose-dependent toxicities associated with CQ usage [107]. Considering the high incidence rate (up to 8 %) of retinopathy resulting from long-term HCQ utilization [108], the American Academy of Ophthalmology has recommended administering CQ at doses <2.3 mg/kg based on the actual body weight of the patient and HCQ at doses <5.0 mg/kg [109]. Therefore, special attention should be paid to the potential ocular side effects of CQ in COVID-19 patients with diabetes mellitus when treating such patients with CQ for COVID-19 infection.

### 6.2 Ototoxicity

It is widely acknowledged that viral infections can contribute to hearing loss. Recent studies have delved into the potential detrimental effects of the COVID-19 virus on the inner ear, necessitating further investigation. Evidence indicates that viruses can attach to the ACE2 receptor, which is expressed in various organ systems, including the cochlea, cochlear nerve, and CNS. By binding to these receptors, the virus may trigger the immune system, potentially resulting in harmful inflammation and damage to the patient's tissue [[110], [111], [112]].

Differences in lower frequencies may stem from infection spread from the nasopharynx, potentially leading to middle ear involvement. However, presently, there is limited published evidence directly linking COVID-19 to tinnitus. Notably, the use of CQ has been associated with ototoxicity, alongside its established retinal toxicity.

Hearing loss in COVID-19 patients could also result from treatment with CQ and HCQ, included in clinical guidelines for COVID-19 treatment in various countries [60]. While retinal toxicity caused by CQ and HCQ is typically monitored through regular ophthalmological exams, changes in hearing and potential ototoxicity have been overlooked. Some reports have described sensorineural hearing loss, tinnitus, dizziness, and other cochleovestibular symptoms following prolonged therapy with high doses of CQ [113]. Both acute and chronic use of CQ has been linked to cochleovestibular ototoxicity.

CQ preferentially accumulates and binds to melanocytes within the inner ear, leading to various injuries such as damage to sensory hair cells, reduction in neural population, loss of supporting cells, and atrophy of the stria vascularis [114]. Notably, the suggested dosage of CQ for COVID-19 infection significantly surpasses the typical dose for malaria treatment, thereby heightening the risk of ototoxic effects. Individuals experiencing hearing loss, tinnitus, or balance issues should be promptly referred for hearing evaluation and intervention after stabilization.

Further research is essential to assess the potential impact of COVID-19 on hearing health and its interactions with COVID-19 treatments on hearing function. Ototoxic side effects need to be taken into account during therapeutic decision-making to ensure patient safety and prevent or manage hearing loss.

## 7 Discussion

COVID-19 has already caused significant public health issues around the world, and developing effective pharmacological treatment options is a critical priority in the fight against COVID-19. But after being tested, some prescription drugs—like CQ—were rapidly eliminated from the predetermined treatment regimen. This article combines the various adverse reactions induced by CQ for treating COVID-19 syndrome under the current study and analyzes the research advances on CQ to treat COVID-19 over the past three years. The conclusions that follow can be made in light of the literature review and discussion that has already taken location:

1. CQ reduces ACE2 expression in the heart, while also inhibiting cardiac K^+^ currents and Ca^2+^ channels, potentially leading to conditions like arrhythmia, cardiac hypertrophy, and heart failure.
2. CQ may induce myopathy and impact CK levels, worsening the symptoms of myasthenia gravis. It can also disrupt lysosomal arrangement and autophagy, potentially exacerbating viral myositis from coronavirus infection.
3. Prolonged CQ treatment for COVID-19 may result in adverse neuropsychiatric effects. It interferes with neurotransmitter function, contributing to neurological and neuropsychiatric symptoms such as epilepsy, coma, and psychosis.
4. CQ usage can disrupt the ecosystem of commensal intestinal microflora, leading to dysbiosis and various gastrointestinal symptoms such as nausea, diarrhea, and vomiting.
5. High doses of CQ administration may induce ocular toxicity, potentially causing retinopathy to develop.
6. In addition to the auditory impacts of the virus, CQ medication may contribute to hearing loss in COVID-19 patients. The risk of ototoxicity could be heightened due to the significantly higher recommended dosage of CQ for treating new coronavirus infections compared to the dose typically prescribed for malaria treatment.

## 8 Conclusion

Numerous research studies and applications have been conducted to explore the unique therapeutic potential of CQ in treating COVID-19. Despite demonstrating some antiviral and anti-inflammatory properties, its mechanism of action and clinical evidence remain inadequately understood, with notable adverse effects and limitations as depicted in Fig. 1. Particularly, its specific target of action remains unclear, and its relevance is not convincingly established.

The aim of this literature review is to investigate the reasons behind the swift discontinuation of CQ for diagnosing and treating new coronavirus pneumonia. Our focus is on evaluating its acceptability and safety for patients afflicted with the illness, specifically examining both its treatment efficacy and the noteworthy side effects. The significance of this work lies in identifying effective therapeutic medications that can mitigate treatment-related and preventive challenges associated with subsequent inflammatory syndromes akin to COVID-19.

Compared to the extant body of research, this article offers a more comprehensive and systematic discussion that delves into the history of CQ usage against SARS-CoV-2 from multiple perspectives. However, certain deficiencies exist within this study, chiefly: firstly, there is no extensive clinical trial data regarding the use of CQ in managing novel coronavirus syndrome, which precludes sufficient support for the results presented in this article. Secondly, obtaining suitable data can prove tremendously challenging and may engender deviations in the findings. Therefore, it is vital to expand the scope of CQ treatment data available from other viral diseases to ensure the validity of future studies and provide more equitable reference points. Additionally, the literature suggests that researching new diseases using existing, classical therapeutic agents holds great promise for future development, serving as valuable reference material for treatments targeting potential future illnesses. The insights contained within this article demonstrate the immense significance and informative value of mitigating and preventing potential future diseases via CQ administration.

## Funding

This work was supported by 10.13039/501100007129Shandong Provincial Natural Science Foundation (ZR2023QH174) and Lin He's Academician Workstation of New Medicine and Clinical Translation at 10.13039/501100008853Jining Medical University (JYHL2019MS18).

## Data availability statement

No data was used for the research described in the article.

## CRediT authorship contribution statement

**Yunlong Jia:** Writing – original draft, Resources, Formal analysis, Data curation. **Wenjie Tian:** Writing – original draft, Visualization, Validation, Supervision. **Yuyao Li:** Writing – original draft, Visualization, Supervision. **Yuyan Teng:** Writing – review & editing, Writing – original draft, Supervision. **Xiaolin Liu:** Writing – original draft, Visualization, Supervision. **Zhengyu Li:** Writing – review & editing, Writing – original draft, Validation. **Mingsheng Zhao:** Writing – review & editing, Supervision, Funding acquisition, Conceptualization.

## Declaration of competing interest

The authors declare that they have no known competing financial interests or personal relationships that could have appeared to influence the work reported in this paper.

## Figures and Tables

**Table:** 

Abbreviation	Definition
CQ	Chloroquine
COVID-19	Coronavirus Disease 2019
SARS-CoV-2	Severe Acute Respiratory Syndrome Coronavirus-2
IL	Interleukin
TNF	Tumor Necrosis Factor
ACE2	Angiotensin Converting Enzyme 2
PH	Potential of Hydrogen
FDA	Food and Drug Administration
EUA	Emergency Use Authorization
CK	Creatine Kinase
CNS	Central Nervous System
HCQ	Hydroxychloroquine
hERG	human Ether-a-go-go-related Gene
